# Association between plasma leptin and cesarean section after induction of labor: a case control study

**DOI:** 10.1186/s12884-021-04372-6

**Published:** 2022-01-14

**Authors:** Whitney Cowman, Sabrina M. Scroggins, Wendy S. Hamilton, Alexandra E. Karras, Noelle C. Bowdler, Eric J. Devor, Mark K. Santillan, Donna A. Santillan

**Affiliations:** 1 Department of Obstetrics & Gynecology, University of Iowa Hospitals & Clinics, 200 Hawkins Drive, 463 MRF, Iowa City, IA 52242 USA; 2 Present Address: Department of Obstetrics & Gynecology, Iowa Methodist Medical Center, 1200 Pleasant Street, Des Moines, IA 50309 USA

**Keywords:** Induction of labor, Fetal intolerance of labor, Cesarean section, Vaginal delivery, Leptin, Obesity, Pregnancy

## Abstract

**Background:**

Obesity in pregnancy is common, with more than 50% of pregnant women being overweight or obese. Obesity has been identified as an independent predictor of dysfunctional labor and is associated with increased risk of failed induction of labor resulting in cesarean section. Leptin, an adipokine, is secreted from adipose tissue under the control of the obesity gene. Concentrations of leptin increase with increasing percent body fat due to elevated leptin production from the adipose tissue of obese individuals. Interestingly, the placenta is also a major source of leptin production during pregnancy. Leptin has regulatory effects on neuronal tissue, vascular smooth muscle, and nonvascular smooth muscle systems. It has also been demonstrated that leptin has an inhibitory effect on myometrial contractility with both intensity and frequency of contractions decreased. These findings suggest that leptin may play an important role in dysfunctional labor and be associated with the outcome of induction of labor at term. Our aim is to determine whether maternal plasma leptin concentration is indicative of the outcome of induction of labor at term. We hypothesize that elevated maternal plasma leptin levels are associated with a failed term induction of labor resulting in a cesarean delivery.

**Methods:**

In this case-control study, leptin was measured in 3rd trimester plasma samples. To analyze labor outcomes, 174 women were selected based on having undergone an induction of labor (IOL), (115 women with successful IOL and 59 women with a failed IOL). Plasma samples and clinical information were obtained from the UI Maternal Fetal Tissue Bank (IRB# 200910784). Maternal plasma leptin and total protein concentrations were measured using commercially available assays. Bivariate analyses and logistic regression models were constructed using regression identified clinically significant confounding variables. All variables were tested at significance level of 0.05.

**Results:**

Women with failed IOL had higher maternal plasma leptin values (0.5 vs 0.3 pg, *P =* 0.01). These women were more likely to have obesity (mean BMI 32 vs 27 kg/m^2^, *P* = 0.0002) as well as require multiple induction methods (93% vs 73%, *p* = 0.008). Logistic regression showed Bishop score (OR 1.5, *p* < 0.001), BMI (OR 0.92, *P* < 0.001), preeclampsia (OR 0.12, *P* = 0.010), use of multiple methods of induction (OR 0.22, *P* = 0.008) and leptin (OR 0.42, *P* = 0.017) were significantly associated with IOL outcome. Specifically, after controlling for BMI, Bishop Score, and preeclampsia, leptin was still predictive of a failed IOL with an odds ratio of 0.47 (*P* = 0.046). Finally, using leptin as a predictor for fetal outcomes, leptin was also associated with of fetal intolerance of labor, with an odds ratio of 2.3 (*P* = 0.027). This association remained but failed to meet statistical significance when controlling for successful (IOL) (OR 1.5, *P* = 0.50).

**Conclusions:**

Maternal plasma leptin may be a useful tool for determining which women are likely to have a failed induction of labor and for counseling women about undertaking an induction of labor versus proceeding with cesarean delivery.

## Background

Maternal obesity is associated with a significant increase in the incidence of miscarriage, congenital anomalies, macrosomia, gestational diabetes, gestational hypertension, preeclampsia and stillbirth [1–5]. Obese women have an increased incidence of pre-existing diabetes and chronic hypertension [2] and are more likely to deliver via Cesarean section [2, 3, 5–9]. Obese women are also more likely to undergo IOL [9, 10]. Induction of labor (IOL) is often undertaken because of prevalent co-morbidities such as preeclampsia, hypertension, or diabetes, but obese women are also twice as likely to require induction for postdates pregnancy (> 41–42 weeks gestation) [11–13]. Between 2012 and 2016, 21% of live-births in the United States underwent IOL; of these inductions, 19.2% ended with delivery by Cesarean section [14].

A variety of factors are associated with IOL success, including Bishop score, maternal age and parity [15, 16]. Importantly, obesity has been reported as an independent risk factor for Cesarean delivery following IOL when multiple factors are evaluated, with the highest risk occurring in women with a BMI of 40 or greater [6, 10]. Obesity has also been described as an independent predictor of dysfunctional labor [11, 12, 17–19]. For example, Vahratian et al. showed that labor progression in obese women was significantly slower than normal weight women in early labor [8], and they tended to have a longer total duration of labor and higher oxytocin requirements [9, 20].

Some studies have suggested that dysfunctional labor occurs because there is a reduction in contractility of the obese uterus due to increased cholesterol deposits in the myometrium [19, 21], however, others have found no difference in spontaneous contractile activity of myometrium or response to oxytocin in vitro, attributable to body mass index (BMI) [22]. Leptin, an adipocytokine with an important role in the regulation of energy homeostasis and other neuroendocrine functions [23–26], is found in higher levels in obese individuals, including obese pregnant women [27–31]. Leptin has been shown to be dysregulated in pregnancy, based on maternal condition. For example, individuals suffering from mild and severe preeclampsia had higher levels of leptin compared to healthy pregnant women [32], where a positive correlation with the levels of the steroid hormone, estradiol was found [33, 34]. Furthermore, leptin is associated with a functional importance during pregnancy due to increasing levels until late second or early third trimester followed by a sharp drop off postpartum [35–37]. Leptin has also been found to exert an inhibitory effect on spontaneous and oxytocin-induced contractions in vitro, with both a decrease in the frequency and amplitude of contractions [37, 38].

Because obese women are at increased risk for failed IOL resulting in a cesarean section, we were interested in the role of leptin in induced labor. More specifically, we and others [39] hypothesize that leptin may play an important role in dysfunctional labor. In this cohort study, we investigate whether elevated plasma leptin levels are associated with the outcome of induction of labor at term.

## Methods

### Patient selection and plasma collection

Coded samples and clinical data were obtained from the University of Iowa Institutional Review Board (IRB)-approved Maternal-Fetal Tissue Bank (MFTB) [40] (IRB# 200910784). Informed consent was obtained by the MFTB from women to provide biological samples and clinical data from their electronic health record (Epic) for future studies. Women were recruited at prenatal appointments during their first trimester; longitudinal samples and data were collected throughout pregnancy. Samples used in this study were collected between March 2010 and February 2015. The University of Iowa IRB approved this study’s protocol (IRB# 201711717) [41]. All local and federal guidelines and assurances were followed. The MFTB provided coded plasma samples and corresponding clinical patient information were obtained for all women who had undergone induction of labor at greater than or equal to 37 weeks gestation and met the following inclusion criteria: induction of labor by any method (misoprostol, dinoprostone, PGE2 gel, oxytocin, artificial rupture of membranes, membrane stripping or Foley balloon placement), complete delivery data in the medical record and availability of plasma sample collected in the third trimester (> 28 weeks). Specific exclusion criteria for this study included multiple gestation, fetal anomalies, augmentation of labor rather than induction of labor, intrauterine fetal demise, and absence of an available plasma sample. Exclusion criteria of the Maternal Fetal Tissue Bank, in general, include HIV+, Hepatitis C+, non-English speaking, inability to provide informed consent, and being less than 18 years of age. Because this study was conducted using previously biobanked samples and corresponding clinical data, no interventions were made in the care of patients. All decisions for medical care including induction and delivery methods were made by the patient and her care team. Blood samples were collected at admission for delivery prior to the beginning of induction. All methods were followed in accordance with this IRB determination and all federal guidelines and assurances.

Blood samples obtained for the MFTB are collected in ACD-A tubes (Becton Dickinson) and are separated into blood plasma and mononuclear cells. Plasma is then aliquoted, snap frozen and stored at -80^o^ C [40]. The MFTB maintains a database to annotate samples with clinical information, which is automatically extracted from the electronic health record and stored in a secure database at the University of Iowa [40]. This database was used to identify women eligible for inclusion in this study. A member of the research team individually verified the clinical information housed in this database for each of the study participants.

From the Maternal Fetal Tissue Bank, all participants that met inclusion/exclusion criteria and had samples available were identified. We were able to retrieve coded 3rd trimester blood plasma samples and clinical data from 174 women who underwent an induction of labor and met inclusion criteria; of these women 59 were delivered via Cesarean section and 115 delivered vaginally.

Coded information on maternal and neonatal characteristics were collected from the MFTB. The data gathered included maternal age at delivery, gestational age at induction, race, parity, body mass index (BMI) (at first presentation to obstetric care), weight gain during pregnancy, indication for induction of labor, method(s) of induction, antepartum/intrapartum complications and characteristics (diabetes, hypertension, preeclampsia, history of Cesarean section, intrauterine growth restriction, Rh isoimmunization, oligohydramnios, chorioamnionitis, presence of meconium-stained fluid, use of epidural/spinal anesthesia during labor), method of delivery (vaginal delivery, vaginal birth after Cesarean [VBAC], operative vaginal delivery, Cesarean section) and indication for Cesarean delivery. Neonatal information including weight, APGAR scores, need for resuscitation at delivery, admission to neonatal intensive care unit, and presence of respiratory distress, meconium aspiration, or administration of neonatal antibiotic therapy was also collected. The reason for the Cesarean section was determined from the medical record as determined by the provider. Data were collected from the medical record at least 6 weeks postpartum.

### Maternal plasma analyses

Leptin levels were measured using the commercially available Human Leptin Instant ELISA (ThermoFisher), which has a sensitivity of 20 pg/mL and a standard curve range of 63–4000 pg/mL. Bicinchoninic acid (BCA) levels were measured using a commercially BCA Protein Assay Kit (ThermoFisher), for the quantitation of total protein, which has a working range of 20–2000 μg/mL. All samples were run in duplicate in a single assay. For all analyses, leptin concentration was normalized to micrograms of total protein in the sample and reported as [picogram/total protein [microgram].

### Clinical definitions

Clinical determinations including fetal intolerance of labor, failure to progress, failure to descend, preeclampsia, intrauterine growth restriction, and diabetes were made by the patient’s healthcare providers. The determinations made at that time were extracted from the electronic health record (Epic) by the research team.

### Statistical analysis

All statistical analyses were performed with SigmaPlot 12.0 software (Systat Software, Inc., California). Logistic regression models were constructed using regression identified and clinically significant confounding variables. In addition, Fisher’s exact tests were utilized for categorical variables. For continuous variables, t-test or ANOVA were utilized. Only data available in the electronic health record or from laboratory measurements were used for analyses. All variables were tested at significance level of 0.05. Based on a recent published leptin effect size of 18 pg/mL in control and preeclamptic plasma, for an power of 90% and α = 0.05 minimally 38 samples per group is necessary [42]. For a parsimonious logistic regression model with 5 covariates minimally 50 samples per group is necessary.

## Results

A total of 174 women underwent induction of labor and were included in this study. Characteristics of the study population are summarized in Table 1. Our study included 59 women who ultimately delivered via Cesarean section compared to 115 women who had successful lOL. A successful IOL was defined as having a vaginal delivery, including operative vaginal deliveries with the assistance of either forceps or vacuum as this was the most frequent utilized definition in a systematic review related to induction of labor. At baseline, the two groups were comparable in terms of maternal age, race, parity, gestational age at induction, weight gain during pregnancy, and use of an epidural during labor. Women with Cesarean section were more likely to have obesity as defined by BMI ≥ 30 kg/m^2^ at the beginning of pregnancy (mean BMI 32 vs 27 kg/m^2^, *P* = 0.0002), preeclampsia (13% vs 2%, *P* = 0.008), lower Bishop score (3 vs 5, *P* < 0.001), lower parity (0.3 vs 0.9, *P* < 0.001) and meconium-stained fluid (35% vs 13%, *P* = 0.002). Method(s) of induction of labor in women ultimately delivered via Cesarean section, was/were more likely to include dinoprostone (65% vs 29%, *P* < 0.001) and Foley balloon placement (26% vs 11%, *P* = 0.03). They were also more likely to require multiple induction methods as defined by use of any two or more methods (93% vs 73%, *P* = 0.008). The indication for Cesarean delivery was failed operative delivery in which the use of forceps and/or vacuum preceded a cesarean delivery (2%), arrest of dilation (failure to progress) (26%), arrest of descent (failure to descend) (31%), and fetal intolerance of labor (52%). Percentages do not equal 100 because the indication for Cesarean section may have included one or more of the above diagnoses (i.e. failure to progress and fetal intolerance of labor).

**Table 1:** Patient Demographics, Pregnancy Characteristics, and Leptin Levels

Variable	Successful IOL (vaginal delivery) ***N*** = 115	Failed IOL (Cesarean section delivery) ***N*** = 59	***P*** Value
Maternal Age at Delivery (mean years, 95% CI)	29.6 (28.6–30.6)	29.9 (28.6–31.2)	0.7
Race: White	87.8% (101)	93.2% (55)	0.7
Race: Black	2.6% (3)	1.7% (1)	0.7
Race: Asian	3.5% (4)	1.7% (1)	0.7
Race: Hispanic	1.7% (2)	0% (0)	0.7
Race: American Indian	0% (0)	1.7% (1)	0.7
Race: Unspecified	3% (3)	1.7% (1)	0.7
Race: Multiracial	1.7% (2)	0% (0)	
BMI (mean kg/m^2^, 95% CI)	27.4 (26.2–28.6)	32.2 (29.9–40.0)	**0.0002**
GA at IOL (mean weeks, 95% CI)	39.8 (39.6–40.0)	39.5 (39.2–39.8)	0.2
Bishop Score (mean, 95% CI)	4.6 (4.1–5.1)	2.6 (2.1–3.1)	**< 0.001**
Parity (mean, 95% CI)	0.9 (0.7–1.1)	0.3 (0.1–0.5)	**< 0.001**
Epidural/Spinal (%, N)	84.3% (97)	83.1% (49)	0.9
Weight Gain (mean kg, 95% CI)	12.7 (11.5–13.9)	13.0 (11.4–14.6)	0.8
**Induction Method**			
Cervidil (dinoprostone) (%, N)	28.6% (33)	64.4% (38)	**< 0.001**
Cytotec (misoprostol) (%, N)	30.4% (35)	44.1% (26)	0.1
Pitocin (%, N)	86.1% (99)	91.5% (54)	0.5
Foley Bulb (%, N)	11.3% (13)	27.1% (16)	**0.03**
AROM (%, N)	55.6% (64)	50.8% (30)	0.7
Nipple Stimulation (%, N)	0.87% (1)	0%	0.7
Multiple IOL Methods (%, N)	73.9% (85)	93.2% (55)	**0.008**
**Indication for IOL**			
IOL PET (%, N)	1.7% (2)	13.5% (8)	**0.008**
IOL AMA (%, N)	11.3% (13)	18.6% (11)	0.2
IOL Fetal Indication (%, N)	18.2% (21)	13.5% (8)	0.5
IOL GDM (%, N)	7.8% (9)	3.4% (2)	0.5
IOL DM I (%, N)	0.87% (1)	6.8% (4)	0.2
IOL DM II (%, N)	0% (0)	1.7% (1)	0.7
IOL CHTN (%, N)	11.3% (13)	15.2% (9)	0.6
IOL gHTN (%, N)	8.6% (10)	3.4% (2)	0.4
IOL Postdates (%, N)	28.6% (33)	30.5% (18)	0.9
IOL PROM (%, N)	6.1% (7)	6.8% (4)	0.8
IOL Elective (%, N)	25.2% (29)	8.5% (5)	**0.03**
IOL Multiple Indications (%, N)	19.1% (22)	18.6% (11)	0.9
**Antepartum/Intrapartum Complications**			
No Pregnancy Complications (%, N)	45.2% (52)	30.5% (18)	0.06
CHTN (%, N)	11.3% (13)	15.2% (9)	0.06
gHTN (%, N)	8.6% (10)	3.4% (2)	0.4
Preeclampsia (%, N)	1.7% (2)	13.5% (8)	**0.008**
DM I (%, N)	0.8% (1)	6.7% (4)	0.07
DM II (%, N)	0% (0)	1.7% (1)	0.2
GDM A1 (%, N)	4.3% (5)	3.4% (2)	0.7
GDM A2 (%, N)	6.1% (7)	3.4% (2)	0.7
Alloimmunization (%, N)	0.8% (1)	1.7% (1)	0.8
IUGR (%, N)	1.7% (2)	0% (0)	0.8
Oligohydramnios (%, N)	7.8% (9)	1.7% (1)	0.2
Cerclage (%, N)	2.6% (3)	1.7% (1)	0.8
Hypothyroid (%, N)	11.3% (13)	8.5% (5)	0.9
AMA (%, N)	12.2% (14)	18.6% (11)	0.4
History of Cesarean Section (%, N)	4.3% (5)	1.7% (1)	0.7
VBAC (%, N)	1.7% (2)	0% (0)	0.8
Operative Vaginal Delivery (%, N)	14.8% (17)	0% (0)	**0.007**
Meconium (%, N)	13.0% (15)	35.6% (21)	**0.002**
Chorioamnionitis (%, N)	7.8% (9)	13.6% (8)	0.4
Gestational age at sample collection	39 5/7 weeks	38 5/7 weeks	0.26
Leptin (pg/ug)	0.3 (0.3–0.4)	0.5 (0.4–0.7)	**0.01**

*BMI* body mass index, *GA* gestational age, *IOL* induction of labor, *AROM* artificial rupture of membranes, *AMA* advanced maternal age (>/= 35 years), *GDM* gestational onset diabetes, *DM I* type I diabetes mellitus, *DM II* type II diabetes mellitus, *CHTN* chronic hypertension, *gHTN* gestational hypertension, *PROM* premature rupture of membranes, *GDM A1* gestational onset diabetes mellitus type I, *GDM A2* gestational onset diabetes mellitus type II, *IUGR* intrauterine growth restriction, *VBAC* vaginal birth after Cesarean

Data are presented as mean with 95% confidence interval or percentage with N. Categorical variables were compared using Chi square. Continuous variables were analyzed using t-Test or ANOVA. α = 0.05

Neonatal characteristics are summarized in Table 2. Women with Cesarean section were more likely to have infants with lower APGAR scores at 1 and 5 min (6 vs 8, *P* < 0.001 and 8 vs 9, *P* = 0.008, respectfully), higher need for resuscitation at time of delivery (54% vs 15%, *P* < 0.001), as well as the diagnoses of respiratory distress syndrome (31% vs 4%, *P* < 0.001) and meconium aspiration syndrome (9% vs 0.9%, *P* = 0.02).

**Table 2:** Neonatal Characteristics

Neonatal Characteristic	Successful IOL	Failed IOL	***P*** Value
Birth Weight (mean grams, 95% CI)	3494 (3395–3593)	3645 (3504–3785)	0.05
APGAR 1 min (mean, 95% CI)	7.9 (7.6–8.2)	6.3 (5.7–6.9)	**< 0.001**
APGAR 5 min (mean, 95% CI)	8.8 (8.7–8.9)	8.4 (8.1–8.7)	**0.008**
Resuscitation (%, N)	14.8% (17)	54.2% (32)	**< 0.001**
Respiratory Distress (%, N)	4.3% (5)	30.5% (18)	**< 0.001**
Neonatal Antibiotics (%, N)	15.6% (18)	22.0% (13)	0.5
Meconium Aspiration (%, N)	0.86% (1)	8.5% (5)	**0.02**
NICU Stay (Mean Days, 95% CI)	1.0 (0–2.2)	1.2 (0.5–1.9)	0.8

*NICU* Neonatal Intensive Care Unit

Data are presented as mean with 95% confidence interval or percentage with N. Categorical variables were compared using Chi square. Continuous variables were analyzed using t-Test or ANOVA. α = 0.05

**Fig. 1:**
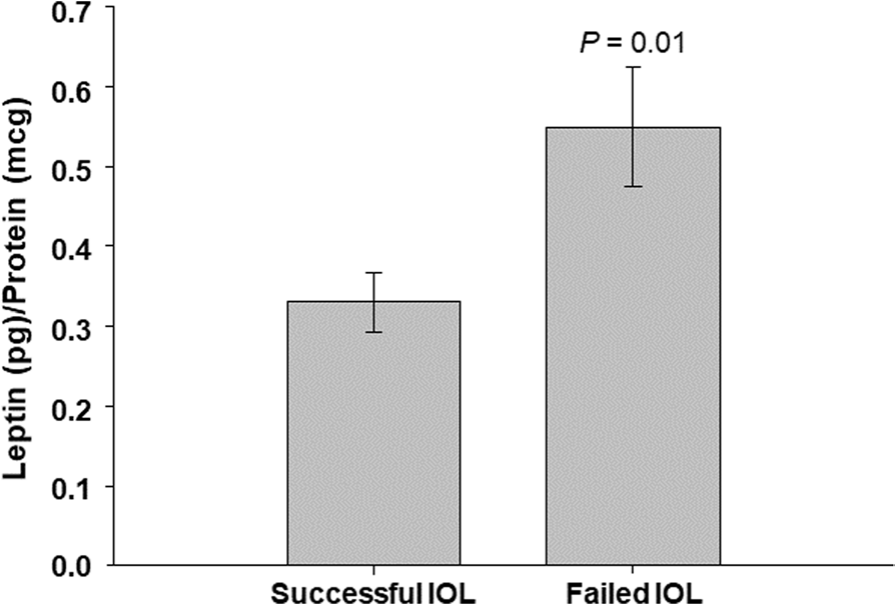
Leptin and successful IOL versus cesarean delivery. Leptin is significantly higher in women with failed IOL compared to successful IOL using a Student’s two-tailed t test (*P* = 0.01)

Maternal plasma leptin levels in women with Cesarean section were higher than those who had a vaginal delivery (0.5 vs 0.3 leptin pg/ug, *P* = 0.01). Figure 1 demonstrates the association between leptin and successful IOL versus Cesarean section delivery, with a 2 tailed Student’s t test *P* = 0.01.

**Fig. 2:**
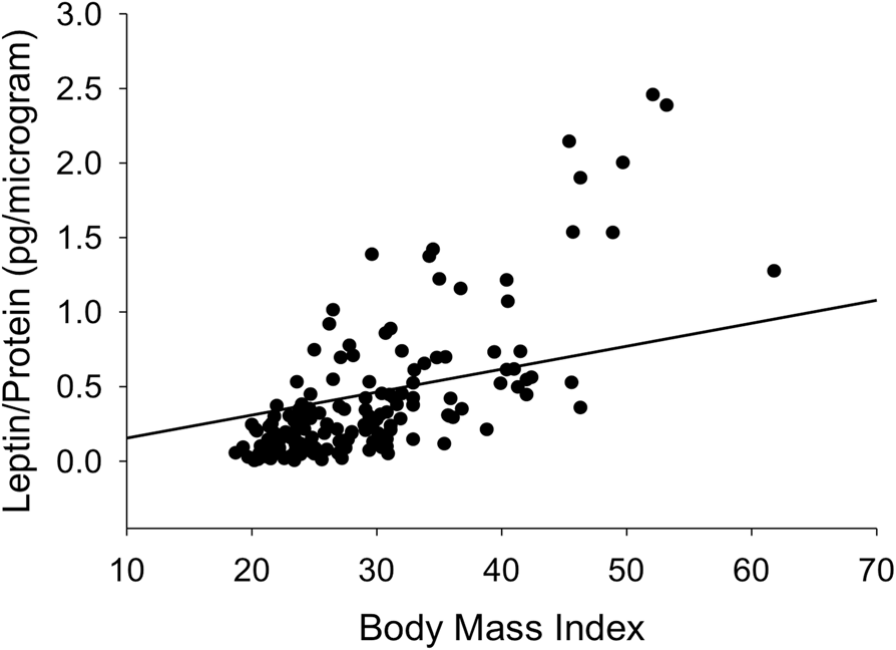
Correlation between Leptin and BMI. We and others demonstrate that leptin and BMI are highly correlated and therefore, cannot be used in the same model (*R*^2^ = 0.73, *P* < 0.001)

**Fig. 3:**
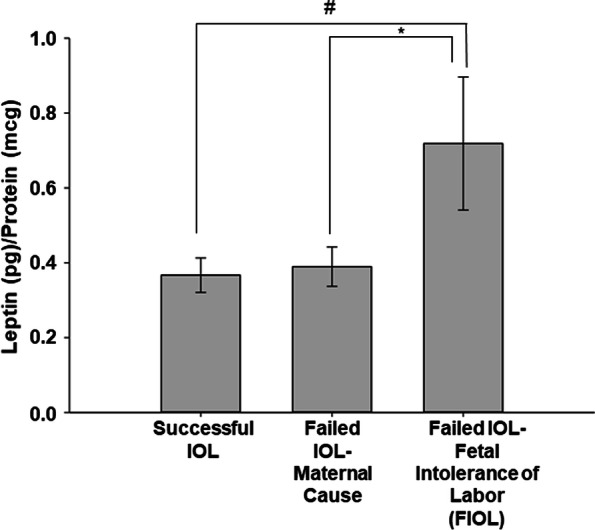
Leptin and prediction of fetal intolerance of labor. The reason for failed IOL was examined in regard to maternal indication versus fetal intolerance of labor. Leptin was significantly higher in those women with FIOL in comparison to those with either successful IOL (#, *P* = 0.01) or failed IOL (*, *P* = 0.03) due to maternal indication

A regression analysis was performed, and the results of the various models are shown in Table 3. Bishop score (OR 1.5, *P* < 0.001), BMI (OR 0.92, *P* < 0.001), preeclampsia (0.12, *P* = 0.01), use of multiple methods of induction (OR 0.22, *P* = 0.008) and leptin (OR 0.42, *P* = 0.02) were the only covariates significantly associated with successful IOL. BMI and leptin were co-linear variables, as shown in Fig. 2. Thus, after controlling for Bishop score and preeclampsia, leptin was still predictive of successful IOL with an odds ratio of 0.47 (*P* = 0.046). Finally, a model using leptin as a predictor for fetal outcomes demonstrated that leptin was also predictive of fetal intolerance of labor (FIOL), with an odds ratio of 2.3 (*P* = 0.03). These models are shown in Table 4 and Fig. 3.

**Table 3:** Logistic Regression Models and Association with Successful Induction of Labor

Model	Bishop Score	BMI	Leptin	Preeclampsia	Multiple IOL Methods
1	1.5 (< 0.001)				
2		0.92 (< 0.001)			
3			0.42 (0.017)		
4				0.12 (0.010)	
5					0.22 (0.008)
6	1.5 (< 0.001)	0.93 (0.004)			
7	1.5 (< 0.001)		0.49 (0.056)		
8	1.4 (< 0.001)	0.94 (0.004)		0.186 (0.046)	
9	1.4 (< 0.001)		0.47 (0.046)	0.16 (0.037)	
10	1.4 (< 0.001)	0.94 (0.008)		0.22 (0.071)	0.28 (0.06)
11	1.4 (< 0.001)		0.45 (0.057)	0.20 (0.058)	0.25 (0.043)

Data are presented as odds ratio with *p* value for each independent variable. The dependent variable for each model is the occurrence of a successful induction of labor

**Table 4:** Linear regression models and association with fetal outcomes

Model	Apgar 1	Respiratory Distress	NICU Days	Fetal Intolerance of Labor (FIOL)	Successful IOL
1	0.80 (0.486)				
2		0.88 (0.850)			
3			0.34 (0.254)		
4				2.3 (0.027)	
5				1.5 (0.50)	1.0 (< 0.0001)

Data are presented as odds ratio with *p* value for each independent variable. The dependent variable for each model is Leptin level

## Discussion

The pathophysiology of obesity in pregnancy is not completely understood but involves a host of environmental and genetic factors. A complex physiologic system is responsible for the regulation of energy homeostasis and the adipocytokine, leptin, is among the key players [23]. Leptin is a 16-kDa polypeptide product of the *LEP* gene (also known as *OB, OBS, LEPD*) in humans, first isolated by Zhang et al. [43] in 1994 and mapped to chromosome 7 in 1995 [44], although an obesity causing mutation in mice was described long before [45, 46]. Leptin is produced primarily by adipocytes and its secretion is highly proportional to body fat content [23, 28, 30, 47]; and more specifically, to the stored amount of lipid in the fat cells of adipose tissue [29]. Obese individuals have been demonstrated to have markedly elevated leptin levels [27–30]. As one of the primary signals from the body’s energy stores, leptin acts on the central nervous system (CNS) to inhibit appetite and promote satiety and energy expenditure [23, 25]. Because obese individuals have high circulating levels, the concept of leptin resistance emerged [24, 25]. Characterized by elevated serum leptin levels and decreased leptin sensitivity, leptin resistance is not completely understood but may represent a fundamental pathology of obesity.

To our knowledge, this is the first-time maternal 3rd trimester leptin has been shown to be associated with failed induction of labor resulting in cesarean section, with an odds ratio of 0.47 (*P* = 0.046). Not surprisingly, leptin levels were associated with increasing BMI. This is consistent with prior data that has shown maternal levels in pregnancy increase linearly with pre-pregnancy BMI [31]. Thus, in future studies to better elucidate the role of leptin, the Cesarean section and vaginal delivery after IOL and groups will need to be better matched for BMI. We hypothesize that increased circulating leptin levels play an important role in the pathophysiology of dysfunctional labor due to an inhibitory effect on uterine smooth muscle, thus significantly increasing the likelihood for Cesarean section delivery. As previously highlighted, prior research has shown that leptin exerts an inhibitory effect on spontaneous and oxytocin-induced myometrial contractions, with both a decrease in the frequency and amplitude of contractions [38]. Leptin also has the ability to inhibit myometrial apoptosis [48] and extracellular matrix remodeling [49], both proposed as essential steps needed for the uterus to develop powerful and synchronous contractions during labor [50–54]. In addition, extracellular matrix remodeling has been implicated in rupture of membranes and cervical ripening [55, 56]. Our results support the concept of a possible metabolic regulation of the human myometrium during pregnancy and labor and highlight one of the proposed mechanisms for dysfunctional labor and failed induction of labor in obese women.

Obesity is well-established as a significant predictor of failed IOL, but induction is often medically indicated for any one of the varieties of pregnancy-related complications that are more common in obese women. The risk for an emergency or unplanned Cesarean delivery in nulliparous women increases proportionally with a patient’s BMI [7] and although the increased morbidity associated with Cesarean delivery following prolonged labor or rupture of membranes is true for all women, the obese population are at greater risk for complications in these settings [57, 58], including higher rates of infection, wound complications, and postpartum hemorrhage [2]. The risk of failed induction and increased rate of complications with cesarean delivery puts the clinician in a difficult position when counseling about induction of labor. Thus, research aimed at identifying factors that are associated with the outcome of induction of labor is clinically important.

Currently, the Bishop score has traditionally been one of the main factors used to predict success of labor induction. The Bishop score assesses position, consistency, effacement and dilatation of the cervix and the fetal station [59]. However, a systematic review of 40 articles found the Bishop score to be a poor predictor [60]. This may be due to the subjective nature of the measurement that can vary between observers [61]. Thus, there continues to be a gap in being able to counsel pregnant women about the possibility of successful induction of labor, especially in the setting of obesity. Additionally, having a better prediction of the mode of delivery can help healthcare providers determine the best healthcare facility for delivery. This can be especially helpful in settings in which there is not 24-h onsite access to a surgeon or anesthesia. To fill these gaps, researchers need to identify a reproducible and objective method to predict cesarean delivery [62]. Objective methods for predicting successful induction of labor may include the use of ultrasound or biomarkers.

Strengths of the study were adequate power to detect a clinically significant difference in outcomes. Also, using the Maternal-Fetal Tissue Bank provides a regulated, maintained, and unbiased platform for clinical research, with consistency established in the acquisition and storage of tissue/plasma samples and clinical information. The consistency in sample collection and handling is critical to biomarker studies. Weaknesses of our study include the study’s retrospective design and relatively homogenous study population which may limit generalizability, but should not bias the measurements of leptin.

## Conclusions

In order for prenatal care providers to be able to identify which patients are ideal candidates for a successful induction of labor, there needs to be a better understanding of the physiological mechanisms involved in this process. Because leptin can suppress uterine contractility, it is not surprising that elevated leptin levels are associated with failed induction. Taken together, this suggests a strong mechanistic role for leptin in the regulation of labor which we are continuing to investigate.

## Data Availability

The data that support the findings of this study are available from the Maternal Fetal Tissue Bank at the University of Iowa but restrictions apply to the availability of these data, which were used under an IRB-approved agreement for the current study, and so are not publicly available. Data are however available from the authors upon reasonable request and with permission of the Maternal Fetal Tissue Bank. Requests for data and/or samples should be made to Donna Santillan, PhD (corresponding author).

## Abbreviations

IOL: Induction of labor
IRB: Institutional Review Board
MFTB: Maternal Fetal Tissue Bank
BMI: Body mass index
FIOL: Fetal intolerance of labor
VBAC: Vaginal birth after cesarean section
pg: Picograms
mL: Milliliter
BCA: Bicinchoninic acid assay
ug: Microgram
GA: Gestational age
AROM: Artificial rupture of membranes
AMA: Advanced maternal age
GDM: Gestational onset diabetes mellitus
DM1: Type I Diabetes Mellitus
DMII: Type II Diabetes Mellitus
CHTN: Chronic hypertension
gHTN: Gestational hypertension
PROM: Premature rupture of membranes
GDM A1: Gestational onset diabetes mellitus Type I
GDM A2: Gestational onset diabetes mellitus Type II
IUGR: Intrauterine growth restriction
NICU: Neonatal intensive care unit
